# Multiple cavernous malformations presenting in a patient with Poland syndrome: A case report

**DOI:** 10.1186/1752-1947-5-469

**Published:** 2011-09-20

**Authors:** Karlo J Lizarraga, Antonio AF De Salles

**Affiliations:** 1 Division of Neurosurgery, David Geffen School of Medicine, University of California at Los Angeles, 10945 Le Conte Avenue, Room 2120, Los Angeles, CA 90095, USA

## Abstract

**Introduction:**

Poland syndrome is a congenital disorder related to chest and hand anomalies on one side of the body. Its etiology remains unclear, with an ipsilateral vascular alteration (of unknown origin) to the subclavian artery in early embryogenesis being the currently accepted theory. Cavernous malformations are vascular hamartomas, which have been linked to a genetic etiology, particularly in familial cases, which commonly present with multiple lesions. Our case report is the first to describe multiple cavernous malformations associated with Poland syndrome, further supporting the vascular etiology theory, but pointing to a genetic rather than a mechanistic factor disrupting blood flow in the corresponding vessels.

**Case presentation:**

A 41-year-old Caucasian man with Poland syndrome on the right side of his body presented to our hospital with a secondary generalized seizure and was found to have multiple cavernous malformations distributed in his brain, cerebellum, and brain stem, with a predominance of lesions in the left hemisphere.

**Conclusion:**

The distribution of cavernous malformations in the left hemisphere and the right-sided Poland syndrome in our patient could not be explained by a mechanistic disruption of one of the subclavian arteries. A genetic alteration, as in familial cavernous malformations, would be a more appropriate etiologic diagnosis of Poland syndrome in our patient. Further genetic and pathological studies of the involved blood vessels in patients with Poland syndrome could lead to a better understanding of the disease.

## Introduction

Poland syndrome is a congenital disorder of unknown etiology characterized by ipsilateral hand and chest wall anomalies, including hypoplasia or absence of the breast and pectoral muscles [1]. Accepted theories point to an early deficit of blood flow to the distal limb, the pectoral region, and even the brain stem via the subclavian artery during week six of gestation [2]. Its etiology to date has involved two hypotheses. One proposes that the underlying ribs on the affected side grow too quickly in a forward growth plane and thus reduce blood flow in the arteries. Another proposes that a malformation of the embryonic blood vessel serving the pectoralis muscle and the arm and/or hand on the same side of the body limits blood flow to these structures. Final proof is lacking, and no genetic evidence exists [2-4].

One case report described a patient with Poland-Möbius syndrome (which includes bilateral abnormalities of the oculomotor and facial cranial nerves) who presented with one cavernous malformation (CM) that was causing generalized seizures, which resolved after its surgical resection [5]. CMs are low-flow vascular hamartomas characterized by endothelium-lined sinusoidal chambers that lack the other mural elements of mature vascular structures and a distinctive multi-lobulated, "multi-berry" appearance on MRI scans [6,7]. They can be asymptomatic in up to 40% of patients or may manifest as seizures or neurological deficits [6,7]. Sporadic and familial forms of CMs have been described, with the latter comprising at least 6% of all cases [6,8]. Multiple lesions are common in familial forms [8,9]. Hereditary forms of cerebral CMs are caused by mutations in one of three genes, *KRIT1 *(*CCM1*), *CCM2 *(*MGC4607*), and *PDCD10 *(*CCM3*), that may have a role in the genesis of vascular endothelial cells. Their critical importance to lesion development is underlined by the observation that at least one of the CCM genes is mutated in most familial CMs [10-12]. The association of Poland syndrome with multiple CMs described in our patient offers support to the vascular theory as the underlying mechanism for this pathological entity, but pointing to a genetic rather than a mechanistic origin of the proposed vascular disruption.

## Case presentation

A 41-year-old Caucasian man was sleeping when he suddenly experienced tonic movement of the right upper limb followed by loss of consciousness and clonic movements of his four extremities, all of which lasted approximately one minute and was self-limited. Our review of his symptoms was negative, including headaches and other neurologic symptoms or signs. His medical history included hypertension and Poland syndrome. His mother had had chronic obstructive pulmonary disease and had a heart attack at age 64, which caused her death. The rest of the patient's family history was negative for neurologic or vascular alterations.

**Figure 1:**
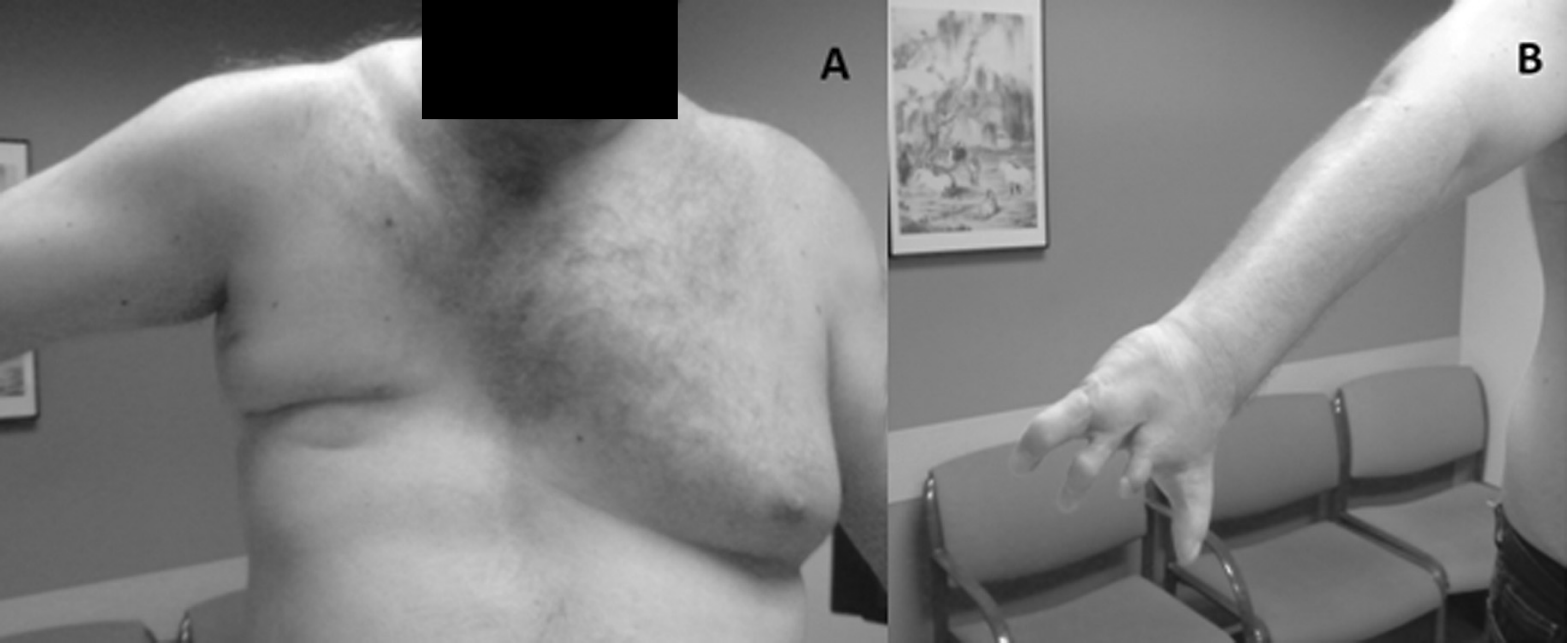
**Features corresponding to Poland syndrome in our patient**. **(A) **Absence of right breast and right pectoralis major muscles. **(B) **Right hand symbrachydactyly.

**Figure 2:**
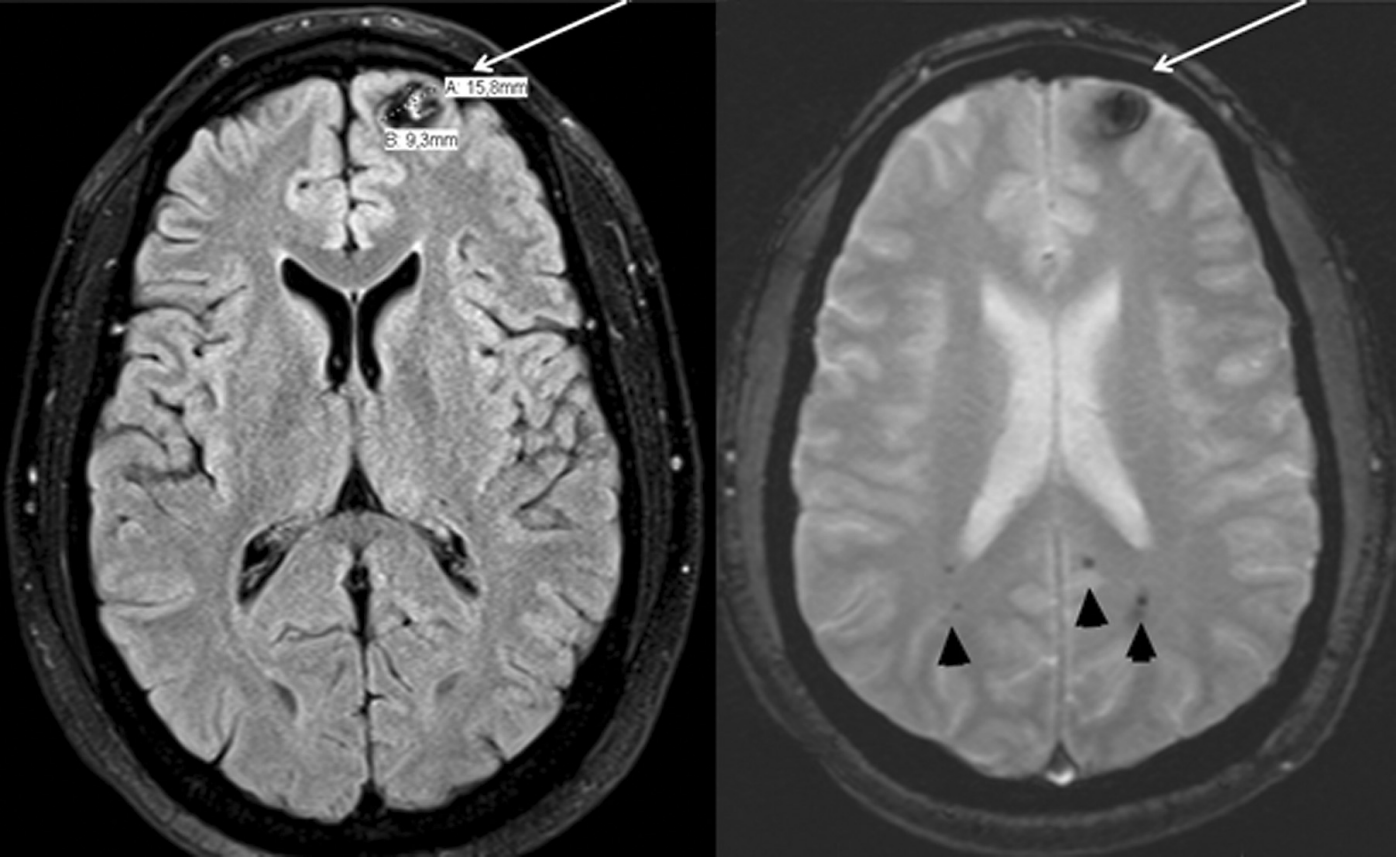
**The patient’s largest cavernous malformation is shown in the left frontal pole. **This lesion has classic signs of hemorrhage (white arrows). More lesions compatible with cavernous malformations in other areas of the brain can also be observed (arrowheads).

**Figure 3:**
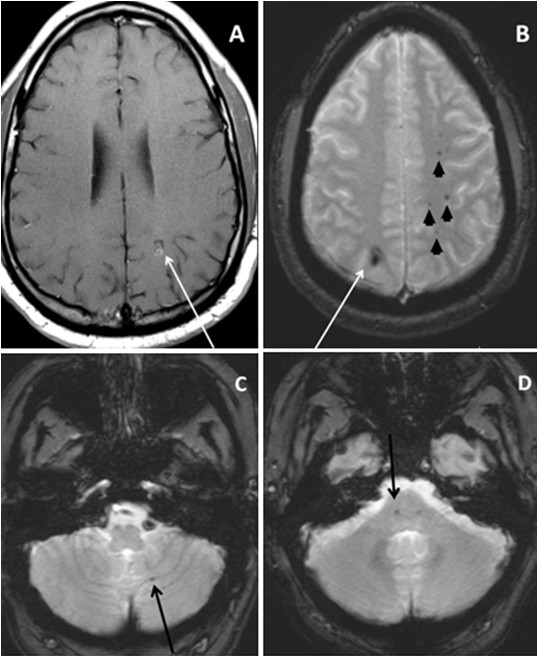
**Multiple cavernous malformations in our patient. (A) **and** (B) **Cavernous malformations in both parietal lobes showing signs of hemorrhage (white arrows). Other multiple cavernous malformations in the brain, predominantly in** (B) **the left hemisphere (black arrowheads),** (C) **the cerebellum (black arrow), and** (D) **the brain stem (black arrow).

A physical examination revealed the absence of his right breast and right pectoralis major muscles (Figure 1A) and right-handed symbrachydactyly, status post-multiple corrective surgeries (Figure 1B), corresponding to Poland syndrome. His neurological examination results were within normal limits. MRI studies showed multiple cerebral CMs, with the largest one, measuring 15.8 mm × 9.3 mm, located in the left frontal pole cortical region, with evidence of recent hemorrhage (Figure 2). Two other CMs located in the right and left parietal lobes also showed evidence of bleeding (Figures 3A and 3B). The majority of the CMs were located in the left hemisphere (Figure 3B). Multiple other cerebellar and brain stem CMs were also noted (Figures 3C and 3D).

The patient presented to our hospital to discuss the possibility of undergoing stereotactic radiosurgery for treatment of the CMs. He had already been started on levetiracetam 75 mg twice daily, which led to good seizure control. Thus, electroencephalographic monitoring and/or telemetry to detect the seizure origin localization, continuing medical therapy, and observation were recommended at that point.

## Conclusion

While the pathogenesis of cerebral CMs on a genetic basis is starting to be better understood, the etiology of Poland syndrome remains uncertain, with an ipsilateral vascular alteration (of unknown origin) to the subclavian artery in early embryogenesis being the currently accepted theory [2,3]. We present the first case report in the literature describing a patient with multiple CMs associated with Poland syndrome, providing further evidence in favor of a vascular disruption during early angiogenesis as its possible etiology.

Our patient presented to our hospital with a secondary generalized seizure, and the corresponding imaging studies showed multiple CMs in his brain, brain stem, and cerebellum. Three CMs showed signs of hemorrhage and it could not be ascertained which one caused his seizure. Thus, we suggested that specialized imaging studies be obtained for seizure localization. If the superficial frontal lesion correlated with the seizure origin, the patient would benefit from its surgical resection; meanwhile, since the seizures were being appropriately controlled with medication, observation was recommended.

Interestingly, the majority of lesions in our patient were found to be located in the left cerebral hemisphere, contralateral to his chest wall and hand defects. A mechanistic external factor could not explain our patient's presentation, because it would not be able to cause an alteration in blood flow to the right upper limb and right chest wall and at the same time to the left cerebral hemisphere. On the other hand, this particular distribution of lesions might be explained if a genetic alteration of the vessels, which occurs in familial CMs, were responsible. The possibility of a coincidental presentation of Poland syndrome and multiple CMs is also worth considering, but it would be more plausible if a single CM were present rather than the multiple and diffuse cerebral vascular compromise in our patient. Any of these proposals, and therefore a better understanding of the disease, could be addressed by conducting pathological studies of the affected limb blood vessels in patients with Poland syndrome and comparing them with anomalies already found in CMs.

## Consent

Written informed consent was obtained from the patient for publication of this case report and any accompanying images. A copy of the written consent is available for review by the Editor-in-Chief of this journal.

## Competing interests

The authors declare that they have no competing interests.

## Authors' contributions

KJL analyzed and interpreted the patient data and the corresponding literature. AAFD was directly involved in the patient's care. All authors were major contributors to writing and reviewing the final manuscript.
